# Demographics, Clinicopathological Profile of Oral Lichen Planus of South Kerala Population: A Cross-Sectional Study

**DOI:** 10.7759/cureus.29688

**Published:** 2022-09-28

**Authors:** Aswathy K Vijayan, Arvind Muthukrishnan, Aparna M Nair, Joyal Jose Baby

**Affiliations:** 1 Department of Oral Medicine and Radiology, PMS College of Dental Science and Research, Thiruvananthapuram, IND; 2 Department of Oral Medicine & Radiology, Saveetha Dental College & Hospital, Chennai, IND; 3 Department of Periodontology, PMS College of Dental Science and Research, Thiruvananthapuram, IND; 4 Department of Prosthodontics, PMS College of Dental Science and Research, Thiruvananthapuram, IND

**Keywords:** reticular, oral lichen planus, hyperpigmentation, erosive, dysplasia

## Abstract

Background:* *Oral lichen planus (OLP) is a chronic mucocutaneous inflammatory lesion of unknown cause. The buccal mucosa is the most frequently affected anatomic site and the lesion is bilateral. The objective of this retrospective study was to investigate the epidemiological and clinical characteristics of 250 OLP patients in South Kerala. This was done by figuring out these patients’ epidemiological and clinical characteristics.

Materials and methods*:* In the present study, patients who reported between September 2018 and December 2021 were selected employing the diagnostic criteria consistent with the WHO classification of OLP. Data of all the patient’s characteristic features were collected and evaluated using the Statistical Package for the Social Sciences (SPSS) software for statistical analysis.

Results: Out of 250 patients, 52% were females and 48% were males. Reticular (n = 145, 58%) and erosive forms (n = 105, 42%) were the two clinical presentations of the lesions that were most consistently observed. The age group of 25-34 years reported the highest number of cases (n = 71, 28.4%). According to the chi-square test, there were statistically highly significant differences between the hyperpigmentation, age, and type of OLP. While 43.2% (n = 108) of cases reported a burning sensation, pain, or soreness, 56.8% (n = 142) of cases were asymptomatic. There were statistically significant differences between the type of OLP and the reported symptoms (p = 0.001). Mild to moderate dysplastic changes were documented in 5.6% during the follow-up period. A successful treatment outcome with topical steroid administration was recorded in the study sample.

Conclusion:* *It was apparent that patients typically have bilateral lesions involving the buccal and labial mucosa, manifesting with varying degrees of oral discomfort. Although patients frequently have more than one variant of OLP, the lesions are typically reticular or erosive in nature. A meticulous follow-up is crucial to determine its malignant change.

## Introduction

Oral lichen planus (OLP) is a mucocutaneous, chronic inflammatory disorder of unknown cause typified by bilateral lesions in various anatomic sites, though they are not necessarily symmetrical [1,2]. While 60% to 70% of patients with cutaneous lichen planus experience oral involvement, 20% to 30% of patients may exhibit only oral manifestations [3]. The buccal mucosa is the most prominently affected area, followed by the tongue, gingiva, labial mucosa, and vermilion of the lower lip. Only 10% of cases have gingival involvement [4]. The age at the onset typically ranges from 30 to 60 years, with a reported female-to-male sex ratio of 1.5 to 3 [5]. Despite the fact that childhood OLP is uncommon, it is documented in the literature that children of Asian descent may be more susceptible to it. This could imply that human leucocyte antigen (HLA-dependent) is associated with a higher incidence of OLP in Asians [6]. OLP is believed to be a T-cell-mediated inflammatory process characterised by type IV hypersensitivity response [7]. The increased production of type-I T helper cytokines, which is genetically induced, is a critical and early stage of OLP. Genetic cytokine polymorphism also appears to be a determining factor. OLP is commonly associated with interferon-gamma. The tumour necrosis factor-alpha is associated with both the cutaneous and oral lesions of OLP [8]. The global prevalence of OLP in the adult population was established to be around 1% to 2%. The estimated prevalence in the Indian population was found to be 2.6% [9]. Due to the significant risk of malignant transformation that the erosive type poses (0.3%-3%), thorough disease surveillance and efficient relapse prevention measures are warranted [10,11]. Direct immunofluorescence (DIF) is a diagnostic adjunct that can be utilised to sustain a diagnosis of OLP even while the diagnosis of OLP is validated by histological and clinical examinations. The most reliable marker for the diagnosis of lichen planus is the occurrence of fibrin deposition at the mucosal-submucosal interface and in cytoid bodies. It may be found either alone or in combination with other immunoreactants, including C3, immunoglobulin (IgM), IgG, and IgA [2,12,13].

Atrophic, reticular, plaque-like, papular, erosive, and bullous variants are the six clinical manifestations of OLP lesions that can be observed. Additionally, they may manifest in a variety of perplexing patterns and forms that mimic other disorders. The classification was then narrowed to three types of OLP: reticular, comprising white plaque-like lesions and papules; atrophic or erythematous; and erosive, including ulcerations and bullous forms [14]. The most prevalent type of OLP is the erosive type, followed by reticular lesions [14]. Orthokeratotic hyperkeratosis, or acanthosis/epithelial atrophy, basal cell degeneration, subepithelial eosinophilic amorphous band, and dense well-defined lymphocytic infiltration in the superficial dermis are histopathological features of OLP. OLP and graft-vs-host disease frequently have similar clinical and histopathologic traits [5]. The malignant transformation of OLP has been found to be between 0% and 10% after a mean review of 1.5-10 years [15]. This depends on how the study was set up and how the samples were chosen.

The most commonly prescribed therapy for OLP is the application of topical corticosteroids since it relieves pain and inflammation. With varying degrees of success, a number of treatments have been explored, including intra-lesional injection, dapsone, retinoids, tacrolimus, and ultraviolet light. Additionally, suggested treatments include laser treatment, cryosurgery, and surgical resection. With protracted steroid therapy, *Candida albicans* are generally encountered in about 37% of OLP lesions. In these cases, a combination of steroid cream and antifungal cream on the skin may help relieve symptoms [6]. The majority of studies have demonstrated that topical corticosteroids are acceptable when administered to mucous membranes for brief periods of time, for up to 6 months. Still, the risk of adrenal insufficiency when corticosteroids are used for a long time, especially for a long-term condition, needs careful and regular monitoring [8]. The oral lichenoid reaction is a prevalent condition that needs to be considered when establishing a differential diagnosis of OLP. They could be regarded as a distinct ailment or an aggravation of an OLP that already existed. Oral and/or cutaneous lesions manifesting lichenoid reactions have been reported after receiving certain medications, such as angiotensin-converting enzyme inhibitors, antidiabetic drugs, and non-steroidal anti-inflammatory medications. It can also be caused by deferred immune-mediated hypersensitivity, leading to allergic contact stomatitis. Symptomless white reticular striae or plaques, painful erythematous or ulcerated patches, and a propensity for unilaterality are the clinical manifestations of lichenoid lesions [2]. Other diseases, such as discoid lupus erythematosus, leucoplakia, and erythroplakia, can also show similar symptoms in the way they look.

OLP evaluation by a multidisciplinary team of healthcare professionals is crucial due to the potential for concurrent lesions in extraoral locations and the threat of oral cancer [16]. As a result, the World Health Organization (WHO) called for more stringent diagnostic standards to produce a more accurate diagnosis of OLP. Van der Meij and van der Waal amended the criteria in 2003 to aid us in achieving unanimity on the diagnostic evaluation of OLP [13]. Several fairly large studies from developed countries have given a detailed description of the clinical and demographic aspects of OLP, but similar studies from developing countries are hard to find [16]. So, this study was done to try to figure out the epidemiological and clinical characteristics of 250 OLP patients in the South Kerala population and to compare and contrast the clinical characteristics with those in previous reports.

## Materials and methods

The present study was initiated after obtaining approval from the Institutional Ethics Committee of PMS College of Dental Science and Research with ethical letter number PMS/IEC/2018-19/40. The demographic information was gathered and described in the pretested case history format after securing informed consent, and a complete intraoral examination was then conducted. The study included 250 OLP patients who attended the Department of Oral Medicine and Radiology and were diagnosed with the condition. These patients were recruited between the time frame of September 2018 and December 2021. The WHO clinical and histological definition of OLP and a set of strict diagnostic criteria proposed by van der Meij and van der Waal were followed [13].

The clinical criteria included were: the occurrence of bilateral, primarily symmetrical lesions, lace-like reticular networks of slightly elevated greyish white lines, erosive, atrophic, bullous, and plaque-type of lesions. The histopathological criteria were: parakeratosis, acanthosis, liquefaction degeneration of the basal layer of cells, presence of lymphocytic infiltration in a band-like arrangement at the level of the papillary layer of the dermis, and absence of epithelial dysplasia.

The study didn’t include people with oral lichenoid lesions that had a known cause like an immune-mediated hypersensitivity reaction to dental restorative materials, a history of systemic diseases, use of tobacco or alcohol, or who were taking drugs like angiotensin-converting enzyme inhibitors or oral hypoglycaemic drugs.

These patients were divided into six groups based on age after the baseline age and gender distribution patterns of the patients were documented. It was also documented how the lesions were distributed throughout the mouth at the time of the initial diagnosis according to symptoms. Clinical variants (reticular, erosive, and atrophic) and anatomic areas affected, including the buccal mucosa, gingiva, labial mucosa, tongue, palate, and floor of the mouth, were identified. In patients who presented with more than one clinical form obvious, the lesions were classified according to the severity of the most severe clinical form. The presence or absence of hyperpigmentation, exacerbating variables of OLP observed by patients or the examiner, and the family history of a first-degree relative with OLP were also recorded. We examined the effectiveness of the treatment during the observation period. Any new systemic illnesses that emerged and any additional cases of lichen planus involvement in the skin or mucous membranes were also reported. To determine the clinical progression of OLP in the study group, we also examined the clinical manifestation of disease in each patient at the commencement and the completion of the follow-up period. When patients gave their consent, some lesions were re-biopsied, particularly in cases where the presence of malignancy or an erosive type prompted concern. A follow-up every 6 months was recommended if possible.

The IBM Corporation was utilised in order to carry out the statistical analysis. The statistical analyses were performed with Statistical Package for the Social Sciences (SPSS) Statistics for Windows, Version 26.0 (IBM Corp., Armonk, USA). The Shapiro-Wilk test was used to determine whether the data follow the normal distribution. Later, descriptive statistics were used to examine the data that had been collected. With a 5% significance level, the chi-square test of association was used to find out if there was a significant difference between the variables.

## Results

Demographic characteristics of the study sample

Two hundred and fifty individuals with a confirmed clinicopathologic diagnosis of OLP were examined. Of these, 120 (48%) were men and 130 (52%) were women, with a male-to-female ratio of 1:1.08. For males, the mean age at initial diagnosis was 35.5 years, and for females, it was 39.1 years. The age range of the patients considered for the current study was 15-74 years. The age groups that had the highest number of patients were those between 25 and 34 years old (n = 71, 28.4%), followed by those between the ages of 35 and 44 (n = 52, 20.8%), those between the ages of 15 and 24 (n = 34, 13.6%), between 45 and 54 years (n = 33, 13.2%), those between the ages of 65 and 74 (n = 32, 12.8%), and those between 55 and 64 (n = 28, 11.2%) years age groups. Table 1 depicts the distribution of the age and gender of the study sample and revealed a statistically insignificant association.

**Table 1:** Age and gender distribution of the study sample n: number

Age		Male	Female	Total	Chi-square test	p-value
15-24	N	17	17	34	4.47	0.48
	% within the age group	50	50	100		
	% within the gender	14.2	13.1	13.6		
	% of total	6.8	6.8	13.6		
25-34	N	37	34	71		
	% within the age group	52.1	47.9	100		
	% within the gender	30.8	26.2	28.4		
	% of total	14.8	13.6	28.4		
35-44	N	20	32	52		
	% within the age group	38.5	61.5	100		
	% within the gender	16.7	24.6	20.8		
	% of total	8	12.8	20.8		
45-54	N	15	18	33		
	% within the age group	45.5	54.5	100		
	% within the gender	12.5	13.8	13.2		
	% of total	6	7.2	13.2		
55-64	N	12	16	28		
	% within the age group	42.9	57.1	100		
	% within the gender	10	12.3	11.2		
	% of total	4.8	6.4	11.2		
65-74	N	19	13	32		
	% within the age group	59.4	40.6	100		
	% within the gender	15.8	10	12.8		
	% of total	7.6	5.2	12.8		
Total	N	120	130	250		
	% within the age group	48	52	100		
	% within the gender	100	100	100		
	% of total	48	52	100		

Further, only 5.6% (n = 14) of the study population disclosed a family history of OLP.

Clinically, reticular lesions were seen in 58% (n = 145) of the patients, followed by erosive forms in 42% (n = 105) of the cases. According to the results of the chi-square test, there is a statistically significant association (p<0.001) between gender and the type of OLP. On comparison, reticular lesions were recorded in 46.2% (n = 60) of females and 70.8% (n = 85) of males. Erosive lesions were found in 53.8% (n = 70) of females and 29.2% (n = 35) of males. Although the majority of patients had multiple oral sites of involvement, the buccal mucosa (n = 150, 60%) and labial mucosa (n = 100, 40%) were the two most frequent sites affected. In relation to the age of the patient and the OLP kinds, the reported hyperpigmentation was found to be significant. The results of the chi-square test of association, which were displayed in Tables 2-3, respectively, showed that there were statistically significant differences between the two groups.

**Table 2:** Association of hyperpigmentation with age **Highly significant n: number

Age		Hyperpigmentation		Total	Chi-square test	p-value
		Present	Absent			
15-24	N	2	32	34	115.74	<0.001**
	% within the age group	5.9	94.1	100		
	% within the hyperpigmentation group	2.1	20.6	13.6		
	% of total	0.8	12.8	13.6		
25-34	N	6	65	71		
	% within the age group	8.5	91.5	100		
	% within the hyperpigmentation group	6.3	41.9	28.4		
	% of total	2.4	26	28.4		
35-44	N	14	38	52		
	% within the age group	26.9	73.1	100		
	% within the hyperpigmentation group	14.7	24.5	20.8		
	% of total	5.6	15.2	20.8		
45-54	N	20	13	33		
	% within the age group	60.6	39.4	100		
	% within the hyperpigmentation group	21.1	8.4	13.2		
	% of total	8	5.2	13.2		
55-64	N	24	4	28		
	% within the age group	85.7	14.3	100		
	% within the hyperpigmentation group	25.3	2.6	11.2		
	% of total	9.6	1.6	11.2		
65-74	N	29	3	32		
	% within the age group	90.6	9.4	100		
	% within the hyperpigmentation group	30.5	1.9	12.8		
	% of total	11.6	1.2	12.8		
Total	N	95	155	250		
	% within the age group	38	62	100		
	% within the hyperpigmentation group	100	100	100		
	% of total	38	62	10		

**Table 3:** Association of hyperpigmentation with the type of OLP **Highly significant OLP: oral lichen planus

Type of OLP	Hyperpigmentation		Total	Chi-square test	p-value
	Present	Absent			
Reticular	87	58	145	70.92	<0.001**
% within the OLP types	60	40	100		
% within the hyperpigmentation group	91.6	37.4	58		
% of total	34.8	23.2	58		
Erosive	8	97	105		
% within the OLP types	7.6	92.4	100		
% within the hyperpigmentation group	8.4	62.6	42		
% of total	3.2	38.8	42		
Total	95	155	250		
% within the OLP types	38	62	100		
% within the hyperpigmentation group	100	100	100		
% of total	38	62	100		

The association between the clinical manifestations of OLP types and the reported symptoms in the study population is shown in Table 4.

**Table 4:** Association with burning sensation and type of OLP **Highly significant OLP: oral lichen planus; n: number

Type of OLP	Symptomatic N (%)	Asymptomatic N (%)	Total N (%)	Chi-square test	p-value
Reticular	23	122	145	105.15	<0.001**
% within the OLP types	15.9	84.1	100		
% within the presence/absence of symptom group	21.3	85.9	58		
% of total	1.2	48.8	58		
Erosive	85	20	105		
% within the OLP types	81	19	100		
% within the presence/absence of symptom group	78.7	13.4	42		
% of total	34	8	42		
Total	108	142	250		
% within the OLP types	43.2	56.8	100		
% within the presence/absence of symptom group	100	100	100		
% of total	43.2	56.8	100		

A total of 108 patients (43.2%) reported symptoms including burning sensations, discomfort, pain, or soreness; while 142 (56.2%) were asymptomatic. Eight percent (n = 85) of erosive lesions and 15.9% (n = 23) of reticular patients experienced discomfort/symptoms.

Clinical follow-up changes

All patients underwent routine follow-up visits, the frequency of which was determined by the clinical characteristic and the requirement for therapy. The disease progression was described as either clinical improvement or exacerbations. Over the follow-up period, if there were any discernible changes in the symptoms and clinical presentation of the lesions it is regarded as clinical improvement. It is considered exacerbations if any changes from asymptomatic to symptomatic lesions, worsening of a symptomatic form, change from reticular to erosive form, or suspicious of malignant transformation. Repeat biopsies were therefore carried out on some of the lesions with the patient’s consent, especially in the more severe cases. Around 5.6% (n = 14) of the study sample exhibited moderate to mild dysplastic changes.

Treatment outcome

The treatment was initiated to achieve total symptom management with few adverse effects. The only topical treatment provided was triamcinolone acetonide 0.1%, which was administered thrice a day for two weeks, and then reviewed, in the symptomatic case. Later based on the clinical improvement, the dose of the drug was tapered eventually. A successful treatment outcome with topical steroid administration was recorded in study sample 1. All the symptomatic patients with reticular (n = 23) and erosive OLP (n = 85) had shown improvement in treatment. Erosive cases need more time for treatment than reticular.

Malignant transformation

There were no incidences of malignant transformation reported over the course of the 28-month follow-up period.

## Discussion

OLP is most generally reported in middle-aged patients between the ages of 30 and 60 [3]. In accordance with numerous other publications, the findings of the current study showed that OLP was frequently observed in women between the ages of 25 and 34 [3,17-19]. The male-to-female ratio by Munde et al. was 1.61:1, with men surpassing women. Typically, it profoundly affects both sides of the buccal and labial mucosa [16]. The mean age of the study sample was comparable to that stated by Munde et al. and different from the majority of the prior studies [16-19]. The mean female age was higher than the mean male age, which was quite resembling the reported findings of Bermejo-Fenoll et al. [18]. The vast majority of patients displayed a stable clinical profile, whereas only a small proportion (5.6%) of patients experienced dysplastic alterations during the period of observation. Carbone et al. have reported comparable results [19]. On the other hand, Thorn et al. found that 17% of spontaneous remissions happen. This may be because they followed up on patients for the longest amount of time, which was about 26 years [20].

In conformity with earlier reported findings, the buccal mucosa was the area most affected [3,21]. These lesions were composed of the typical reticular, erosive, and atrophic forms. Nonetheless, the atrophic forms were excluded during the selection process of the study sample based on the eligibility criteria. The most prevalent type was the reticular form, which was closely followed by the erosive form, consistent with other findings [3,21,22]. The findings of Chainani-Wu et al. were compatible with the incidence of reported erosive lesions in the current study [17]. Xue et al. stated that the reticular lesions showed a substantially larger female preponderance, which was in contrast to the present observations [14]. However, men were more likely to have the reticular type of OLP than women, as reported by Munde et al. and Chainani-Wu et al., which was consistent with our study findings [16,17].

Reticular lesions are frequently asymptomatic, necessitating only routine observation to detect any changes in their clinical status. Erosive or ulcerative OLP, on the other hand, is frequently accompanied by pain and burning sensations [1,18,23]. In contrast, Ingafou et al. reported that oral discomfort was evident in more than 60% of the study group with reticular OLP [15]. According to Munde et al., oral soreness or irritation was observed in 67% of cases [16]. But according to the current study, only 43.2% of the patients had symptoms, which were primarily burning sensations. Prior studies reveal that pain was the most commonly reported symptom overall, although there was also evidence of bleeding, swelling, irritation, and burning [14]. According to the current findings, oral mucosal hyperpigmentation was shown to be present in 38% of cases and was particularly noticeable in a reticular form. Even though it was statistically insignificant, Chitturi et al. observed a greater incidence of pigmentation (67.24%), which could be related to the inclusion of smokers in their study group [24]. Racial and skin-type differences in the local population may also be linked to variations in hyperpigmentation [25]. The pigmentation, which ranged in colour from black to brown and was either patchy or broad, was most noticeable on the buccal mucosa. A few more Indian studies also reported similar results [3,16,24]. The main reasons for hyperpigmentation could be changes that happen after inflammation and repeated episodes of OLP and their healing [24].

Several familial cases of OLP have been described, suggesting that genetic predisposition contributes to the pathophysiology of the disease [23]. While the family history of OLP reported in the current study was similar to that reported by Bermejo-Fenoll et al., it was in contrast to the findings of Munde et al. [16,18]. Although epithelial dysplasia is the gold standard for assessing the risk of oral malignant transformation, podoplanin and adenosine triphosphate (ATP)-binding cassette G2 subfamily protein expression in patients with OLP may be used as biomarkers for risk evaluation. Because there is a lot of difference between and within observers when it comes to interpreting the presence and degree of epithelial dysplasia, immunohistochemistry staining may be more accurate than histopathologic evaluation of epithelial dysplasia for figuring out the chance of malignant change in OLP [26].

In a meta-analysis by Zhou and Vieira, the tumour necrosis factor (TNF-308 G/A) polymorphism was proposed as a possible genetic biomarker for OLP [27]. Yet another genetic predisposing indicator for the development of oral squamous cell carcinoma (OSCC) from OLP is the polymorphism of codon 72 of the p53 gene. Additionally, the proline allele was deemed to be a risk factor since arginine is altered to proline in the protein sequence due to this polymorphism [28]. We can corroborate the chronic nature of this oral condition based on the assessed time of follow-up in our patients, with none of the lesions demonstrating a malignant change. According to Murti et al. and Munde et al., epithelial dysplasia was found in 4% and 3% of cases, respectively, which was in agreement with the current findings [16,29]. Stress, certain foods, sharp dental cusps, systemic diseases, poor oral hygiene, sunlight, and the flu were all things that made the disease worse [14].

OLP lesions typically have protracted clinical manifestations with alternating periods of exacerbations and remissions. During the exacerbation phase, there is an upsurge in erythematous or ulcerated areas as well as in the magnitude of discomfort experienced by the patient [19,23]. According to Tovaru et al., OLP symptoms also worsen in moments of increased emotional turmoil and/or anxiety [22]. The results of Xue et al. were contradicted by the findings of the present study since no indication of a malignant change was identified in the sample, but were consistent with the findings of Munde et al. [14,16]. Murti et al. remarked that investigations conducted in hospitals accounted for the majority of reports of malignant change in OLP. He further stated that only 0.3% of patients in a population-based, 10-year prospective study underwent malignant transformation [29]. Furthermore, van der Meij and van der Waal demonstrated that, among numerous instances, only lichenoid lesions develop into malignant tumours [9,13]. The heightened risk period is between 3 and 6 years following OLP diagnosis. However, the median time between OLP and cancer diagnosis extends from 20.8 months to a whopping 10.1 years [2]. WHO researchers are still trying to figure out what makes OLP a condition that could lead to cancer [5,11]. An increased vulnerability to carcinogens in the erosive type of lichen planus raises the possibility of malignant alterations in these lesions. OLP-associated OSCC progresses differently than squamous cell carcinoma as a whole. Squamous cell carcinomas on the back of the tongue are extremely uncommon, constituting less than 5% of all oral carcinomas. However, malignant transformation of OLP in this site is rather common, and some experts have hypothesised that this region is a substantial risk factor. Chbicheb et al. found that the malignant change of OLP seemed to have nothing to do with external risk factors [11].

Topical corticosteroids are the most effective and versatile treatment for OLP [19]. In the current study, management with triamcinolone alleviated the signs and symptoms of OLP and facilitated ulcer healing and erythema. In order to improve quality of life, treating OLP symptoms is crucial. Systemic corticosteroids should only be used under strict medical supervision. The severity of the discomfort, the patient’s general health, and any challenges with compliance to therapy play a role in the treatment decision. Stress is one of the etiological factors associated with OLP, Given that OLP is a chronic and painful condition, patients received psychological care when required. The study sample did not receive any other specific therapy; instead, only the clinical manifestations based on symptoms were addressed. We saw a good short-term response to topical therapy, which was in line with what Chainani-Wu et al. found [17].

It has been hypothesised that certain *C. albicans* strains can stimulate the production of the carcinogen N-nitroso benzyl methylamine. Additionally, dietary changes, immunosuppression spurred on by symptoms, and therapy could enhance malignant transformation. These lesions are frequently well-differentiated from OSCC histologically [2]. Although a lot of studies indicate a dysregulated immune function, which permits the plausibility of autoimmunity, there has not yet been established proof of autoimmunity in OLP. Diverse microorganisms have been explored for possible involvement in OLP. The most common assumption for OLP linked to the hepatitis C virus (HCV) is autoimmunity [12]. OLP in HCV-positive patients may require a distinctive genetic background, as indicated by a notable topographical heterogeneity in the association between OLP and HCV [5]. A higher prevalence of human papillomavirus (HPV) infections within erosive lesions may also be fostered by the compromised epithelium of the oral mucosa. The greater risk of HPV in OLP observed could be attributed to the immunosuppression from long-term steroid therapy resulting in an amplification of HPV proliferation. According to the meta-analysis by Shang et al., OLP with HPV infection was more common in Asia than in Europe [30]. In this study, fungal overgrowth of normal oral flora leading to candidiasis was infrequent and was associated primarily with the use of topical corticosteroids. Topical antifungal medications controlled this occasional problem. Sharp cusps and improperly fitted prostheses/dental restorations should be inspected for and ruled out as potential sources of oral irritation or mechanical trauma as they may worsen OLP lesions. The maintenance of good oral hygiene is crucial. It is significant to note that some patients report a decrease in oral hygiene practices as a result of the pain and discomfort associated with OLP, triggering a vicious cycle [5].

A thorough search of the literature reveals that there are only a relatively few epidemiological studies of OLP documenting the clinical profile of the Indian population. The current study is exceptional in that it is particularly distinctive as a prospective clinicopathological study that establishes the patient profile, clinical characteristics, and treatment response of the South Kerala population. Although lesions in the tongue, gingiva, palate, and floor of the mouth were frequently described, the study sample only showed lesions in the labial and buccal mucosa. Atrophic OLP was not mentioned either. These differences might be because of the strict criteria for inclusion, which ruled out systemic disorders, drinking alcohol, chewing tobacco, smoking, and drug-related lichenoid reactions.

There are no clear diagnostic standards for OLP that are widely accepted. According to van der Meij’s approach, OLP cases in this investigation that had unilateral lesions on a clinical level and epithelial dysplasia histopathologically were excluded. Even van der Meij and van der Waal. acknowledged that the use of these criteria would prevent some people who could truly have the condition but do not fit the rigid criteria from participating. This difference makes it hard to tell OLP from related lesions, especially oral lichenoid lesions. Both conditions can lead to cancer, so it is very important to know the difference between OLP and lichenoid reactions [9].

The study limitations include a small sample size and the predisposing factors were not completely studied and could be included in further research.

## Conclusions

The histopathologic and clinical findings of the present study indicate that OLP were primarily diagnosed in the adult age group. It was apparent that patients typically had bilateral lesions involving the buccal and labial mucosa, manifesting with varying degrees of oral discomfort. Although patients frequently had more than one variant of OLP, the lesions were typically reticular or erosive in nature. Hyperpigmentation was predominantly observed in reticular lesions of OLP. The lesions are often of a reticular or erosive nature, despite the fact that individuals frequently have more than one type of OLP. Regular follow-up of patients with OLP is highly recommended due to the high malignant transformation potential of such lesions.
